# Estimating historic seabed carbon disturbance by port dredging and aggregate extraction in NW Europe

**DOI:** 10.1371/journal.pone.0349191

**Published:** 2026-05-27

**Authors:** Ellie Maynard, Zoë A. Roseby, James D. Scourse, Sophie L. Ward, Callum Roberts, Ruth H. Thurstan, Ciarán McLaverty

**Affiliations:** 1 Centre for Ecology and Conservation, University of Exeter, Cornwall, United Kingdom; 2 Department of Earth and Environmental Sciences, University of Exeter, Cornwall, United Kingdom; 3 School of Ocean Sciences, Bangor University, Wales, United Kingdom; University of Maryland Center for Environmental Science, UNITED STATES OF AMERICA

## Abstract

The extent to which different human activities disturb seabed carbon, the largest long-term organic carbon reservoir on the planet, is poorly understood. Research to date has focused primarily on bottom trawl fisheries, but industries that target and extract marine sediments are likely to disturb significant masses of sedimentary organic carbon. This can lead to its remineralisation and reduce the capacity of the ocean to absorb atmospheric CO_2_. In this study, we combine archival documents, industry records, and published seabed substrate data, to estimate historical disturbance of sedimentary organic carbon on the Northwest European Shelf (NWES) from port dredging activities and marine aggregate extraction. Monte Carlo simulations were used to provide probabilistic estimates of annual carbon disturbance and associated upper and lower bounds (5th and 95th percentiles, respectively). Based on annual Monte Carlo simulations run between 1995−2021, our results suggest that port dredging over the shelf area disturbed 2.2 ± 0.9 Mt organic carbon year^-1^. In the case of marine aggregate extraction, simulations run between 1955−2022, suggest that marine aggregate extraction disturbed 0.4 ± 0.3 Mt organic carbon year^-1^. At a country scale, analysis of activities in UK waters suggest that organic carbon disturbance from port dredging and aggregate extraction activities are approximately three orders of magnitude lower than published estimates of disturbance by bottom trawling. Nevertheless, historical and contemporary port dredging and aggregate extraction present substantial and spatially concentrated sources of anthropogenic carbon disturbance that have not been systematically quantified across the Northwest European Shelf until now. These findings therefore address an important knowledge gap and have the potential to inform marine management and conservation strategies aimed at minimising organic carbon loss from the seabed.

## Introduction

Continental shelf sea sediments are a primary sink of organic carbon in the ocean [1]. Organic carbon accumulates in marine sediments, sourced from either terrestrial environments or marine environments [2,3]. Despite forming around 7% of the global ocean by area [4], sediments on continental shelves contribute up to 40% of inorganic and 80% of oceanic organic carbon accumulation [5]. Shelf sediments therefore play a critical role in carbon accumulation and sequestration [6], absorbing ~0.2 petagrams (Pg) of carbon annually across the globe [7].

When seabed sediments are physically disturbed, buried organic carbon can re-enter the water column as carbon dioxide via remineralisation [8]. Remineralisation rates are affected by several factors, including disturbance intensity, depth of sediment disturbance, oxygen exposure time [9,10], the source of the organic matter [11,12], and its reactivity [13]. Carbon remineralisation also alters ocean chemistry through acidification [14]. It has the potential to impact atmospheric CO_2_ concentrations, by reducing the capacity of the ocean to absorb atmospheric carbon dioxide [14,15], or by increasing CO_2_ release to the atmosphere [16].

Shelf sea sediments, typically in waters less than 200 m deep, are particularly vulnerable to disturbance from human activities due to relative ease of access [17]. Quantifying the risk of human disturbance to seabed carbon stores has recently gained much scientific and management interest, mainly focussed on the bottom trawl fishing industry [13,14,16,18–20]. There are additional human activities that disturb shelf sea sediments [6], with limited understanding of their impacts on sedimentary carbon. This represents an obstacle to understanding and mitigating their climate impacts [17,21,22].

Dredging of ports, harbours, and navigational access channels (hereafter collectively referred to as port dredging) and marine aggregate extraction are extractive industries characterised by the removal, or redistribution, of sediment from the seabed using specialised dredging equipment. In the case of ports, sediments are dredged either via capital or maintenance dredging, with the extracted material generally disposed at-sea depending on national and international guidelines [23]. Capital dredging is typically undertaken ahead of engineering works (e.g., for port, dock, canal, and marina construction), whereas maintenance dredging ensures navigational depth and prevents siltation of ports and waterways. Mechanised steam dredgers were first built in Sunderland (UK) in 1797 [24], and ports such as Hamburg have been dredged since the 16^th^ century [25]. Marine aggregate extraction generally targets coarse sediments, such as sands and gravels [26], that are used in the construction industry. Although an industry for marine aggregates extraction can be traced back to the 1910s in the United Kingdom, its expansion to large scale commercial operations took place in the 1960s [27].

The physical and biological impacts of marine aggregate extraction are relatively well documented in Europe [28–30], as are the biological impacts of maintenance dredging [31,32], but very little research has investigated the impacts of these industries on shelf sea carbon stores. As both industries are widespread and longstanding, they may have significant implications for our understanding of anthropogenic impacts to marine carbon stocks, and assessments of regional and international carbon budgets. In this study, we provide first-order estimates of seabed organic carbon disturbance by these extractive industries, using the Northwest European Shelf area as a case study due to a relative abundance of available data. We gather data from archival and contemporary sources to quantify the spatial distribution and masses of sediment extraction then estimate the associated carbon disturbance over multidecadal timescales. Finally, we contextualise our estimates of carbon disturbance by comparing our results with other shelf-seabed impacting industries and consider the relative importance of aggregate extraction and dredging on marine sedimentary carbon stocks.

## Materials and methods

### Study area

**Fig 1:**
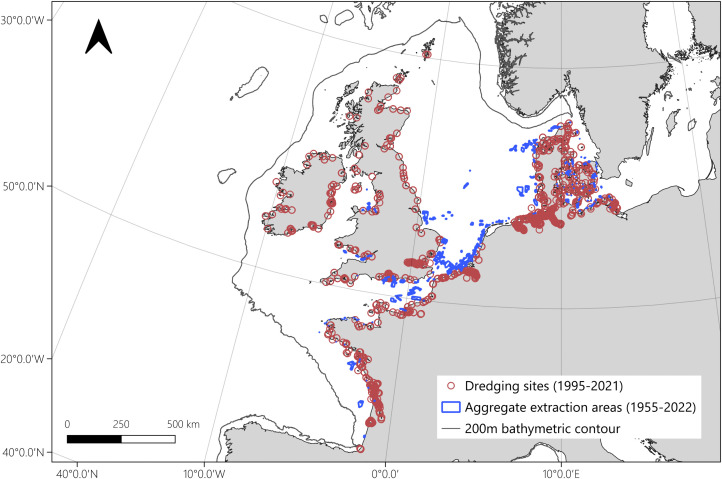
Study area and location of sediment extraction industries. Red circles indicate the location of port dredging events (sourced from EMDODnet), and blue polygons represent marine aggregate extraction licence areas (from EMODnet, ICES reports, and UK public records). 19^th^ century port dredging locations identified in the UK are shown chronologically in Fig 5. The basemap in this figure is in the public domain (available from https://www.naturalearthdata.com/). Projection: LAEA Europe EPSG: 3035.

The Northwest European continental Shelf covers 1.11 million square kilometres, the majority of which is less than 200 metres deep [33] (Fig 1). The area is relatively well studied in terms of seabed biological communities [6] and subtidal sedimentary carbon stocks [1,33,34]. Deposits of coarse sand and gravel are widespread and formed during the advance of grounded ice sheets during the Last Glacial Maximum and reworked during post glacial sea level rise [35]. The Northwest European Shelf is important for fisheries, construction aggregate extraction, marine renewable energy sites, oil and gas extraction, and maritime transport [6,36]. For the purposes of this study, we included port dredging and aggregate extraction data from Belgium, Denmark, France, Germany, the Netherlands, and the United Kingdom, given their relative availability.

### Data sources

Extractive industry data were sourced from parliamentary papers, industry reports, and scientific literature (Table 1). In terms of port dredging, data for the UK were available sporadically between 1834–1845 and 1883–1903 from various archival records. In the earliest of these records, some volumes of sediment were provided only as totals over five-, ten-, or twenty-year periods, while others were listed as volumes extracted without specifying over which years the extraction took place. For example, in the Returns to Parliament from Port and Harbour Authorities (1903), ports and harbours in the UK were requested to report any dredging works that had taken place in the last twenty years, without a standardised reporting method. This meant that the information provided by individual authorities varied in detail and quantity.

**Table 1:** Sources of information used to map extractive industries and calculate extracted sediment masses and organic carbon disturbance across the Northwest European Shelf (NWES) by industry.

Industry	Period	Area	Source
Port and harbour dredging	1798 -1850	UK	Skempton [24]
	1834-1845	UK	First report of the Tidal Harbours Commission [37]
	1883-1903	UK	Returns to parliament from port and harbour authorities [38]
	1995-2021	NWES	EMODnet [39]
Marine aggregate extraction	1955-1972	UK	The Crown Estate, Returns and Royalties [40] & Report to foreshores department [41]
	1970-1977	NWES	Working Group on Effects on Fisheries of Marine Sand and Gravel Extraction (ICES) [42]
	1979-1985	NWES	De Groot [43]
	1989-2000	NWES	Velegrakis et al. [44]
	1990-2022	NWES	EMODnet [39]

For the period 1995–2022, annual data were available for all countries from the European Marine Observation and Data Network (EMODnet) (https://emodnet.ec.europa.eu/en) [39].

Data sourced from EMODnet were subject to some temporal gaps, as in some years data were provided by only a single nation, with a much lower number of records provided in that year relative to others from the same country. Examples of this included port dredging records between 1985–1995 (provided only by UK), and aggregate extraction records between 1985–1989 (Germany, only). Given that annual estimates from a single nation would represent a large underestimate at shelf scale, we excluded those years from our analysis.

### Port and harbour dredging

In the case of port dredging in the UK and Ireland prior to 1903, records typically evidenced the occurrence of works, rather than indicating volumes or masses of sediment removed. Amounts of extracted sediments listed in tons (t) (1016 kilograms), m^3^, or cubic yards were converted to kilograms (kg). Conversions were based on an average density of marine sediment estimated as 1700 kg/m^3^ [45]. An average density was used as sediment type or dry bulk density were often not listed in the original dataset. In these early records, 63 of the 117 ports provided the total monetary cost of dredging works completed at that location. Of these, six ports provided both the costs and the volume of sediment removed. We utilised these six data points to calculate an average cost per kg of sediment removed in years prior to 1903 (S1 Table). This value was then used to derive the estimated mass of sediment removed in cases where only the monetary cost was listed (n = 57) (S2 Table). In cases where neither amount removed or cost of works were listed, but we could confirm that dredging took place (n = 54), an extra step was built into the Monte Carlo simulation (see ‘Quantifying carbon disturbance’ section) to incorporate uncertainties regarding masses of sediment removed into our estimations.

The 63 estimates of sediments removed from ports prior to 1903 were log transformed and then tested for normality with a Shapiro-Wilk test (W = 0.97, p = 0.198). The log-transformed dataset was normally distributed (p > 0.05) and had a mean of 7.2 and standard deviation 0.69 (both reported on a log10 scale). This provided the required parameters to generate a representative, random normal distribution used in the Monte Carlo simulation for resampling, to estimate likely potential masses of sediment extraction in cases where details about masses of sediment removed or cost of dredging works was missing. These estimated sediment masses were back transformed to a base 10 scale before being inserted into the equation used for estimating organic carbon disturbance (see ‘Quantifying carbon disturbance’ section).

Additional impacts to sedimentary organic carbon are expected from the dumping of sediment at sea following port and harbour dredging events, mainly from the resuspension and smothering of seabed sediments at disposal sites. We have not investigated expected impacts from dumping of dredged sediments at sea in this analysis. This is due to limited empirical evidence regarding the biogeochemical fate of smothered sediments, as well as uncertainties surrounding the transport dynamics and subsequent resuspension-transport-resettlement pathways of dumped sediments.

### Marine aggregate extraction

Spatial information describing aggregate extraction areas licensed, and the masses or volumes of sediment extracted from them, after 1990 were available from EMODnet for all countries except Ireland, where information was limited to masses or volumes of sediment extracted. These data were combined with historical aggregate extraction areas provided by ICES reports (e.g., Belgium and the Netherlands) or in some cases public records (e.g., the UK). However, historical spatial data (pre-1990) were not available for some regions, e.g., France, Germany, and Denmark. Due to lack of available information regarding typical organic carbon content, we excluded historical records of maerl extraction from France, Ireland, and the United Kingdom (n = 10) and 61 records of non-aggregate sediment extraction dated between 1881 and 1980 (e.g., tin prospecting, clay, shingle, trial boreholes) from the UK that lacked information about the masses of sediment removed. All marine aggregate extraction areas were digitised using QGIS (Version 3.32.3).

### Quantifying carbon disturbance

To infer how extracted sediment masses relate to seabed carbon disturbance, we analysed sediment organic carbon data from a comprehensive database (EURO-CARBON [46]) of sediment total organic carbon content as dry weight percent (%TOC) from samples collected across multiple habitats in European regional seas (S1 Fig). To ensure that the %TOC data represented the types of sediments targeted by extractive industries, we excluded samples in the database taken from seagrass and saltmarsh habitats. This allowed us to generate %TOC distributions that were representative of sediments targeted by port dredging and aggregate extraction areas on the Northwest European Shelf. This approach was considered to provide more realistic estimate of organic carbon disturbance than would be possible with coarser resolution global models of sedimentary organic carbon density [1], or with regional models of TOC content that do not cover our entire study areas [33,34].

Given that port dredging is generally confined to estuarine, coastal, and inshore areas, we also excluded sediment samples recorded in areas above mean high water and more than 5 km offshore on the shelf (9920). This confined samples to the definition of coastal and inshore areas made by Smeaton et al. [34]. In contrast, marine aggregate extraction areas overlapped poorly with sediment samples in the database (n = 85). Due to the specific substrate types targeted for marine aggregate extraction (i.e., sands, coarse grained, and mixed sediments), we were able to generate representative %TOC content distributions based on the overlap between sediment distribution maps and samples within the EURO-CARBON sediment database [46].

We first overlaid the extraction areas with EMODnet sediment distribution maps, which confirmed sands, coarse and mixed grained sediments were key target substrate types. We then matched any samples from the shelf that overlapped with these sediment classes, resulting in separate %TOC distributions for sand (n = 7262), coarse grained sediments (n = 720), and mixed sediments (n = 273).

Using R (Version 4.3.1), we applied a Monte Carlo simulation with 10,000 runs to estimate annual carbon disturbance using the following:


$$\begin{array}{c} \text{kg of sediment disturbed }*(\%\text{ TOC content }/\text{ 100})\;=\text{ kg of organic carbon disturbed} \end{array}$$

To determine the number of runs required in the Monte Carlo simulation for output convergence, we ran 10 independent iterations of the simulation with 100 runs, and then 10 iterations with 500 runs. We repeated this process, with increasing numbers of runs, until the simulation output stabilised (S2 Fig).

In each simulation run, a %TOC value was randomly selected from the relevant generated distribution, based on the sediment type being extracted in the case of aggregate extraction, or the coastal sediment sample dataset in the case of port dredging. The repeated simulations generated a probabilistic distribution of annual carbon disturbance values, allowing us to quantify ‘most likely’ estimates (reported within text as the mean output ± standard deviation calculated from the Monte Carlo simulation outputs over the specified range of years).

Where masses of sediment disturbance were missing from pre-1903 port dredging events in the UK, kg of sediment disturbance was also randomly selected in each iteration from the generated (log-scale) normal distribution (see Materials and Methods, ‘port and harbour dredging’ section). Each randomly selected mass was back-transformed to a base 10 scale before being used in the equation to estimate organic carbon disturbance. This ensured all results from the Monte Carlo simulation method remained in kg units. Unlike our 20^th^ and 21^st^ century country-level and shelf-wide results, carbon disturbance from pre-1903 port dredging events was first estimated at the port level and then summed to generate a national UK wide estimate. As a result, these historical UK estimates are reported in-text without an associated measure of standard deviation. Uncertainties around these estimates are instead presented in Fig 5, in the same way as the rest of our results, shown with the 5^th^ and 95^th^ percentile estimates.

**Fig 2:**
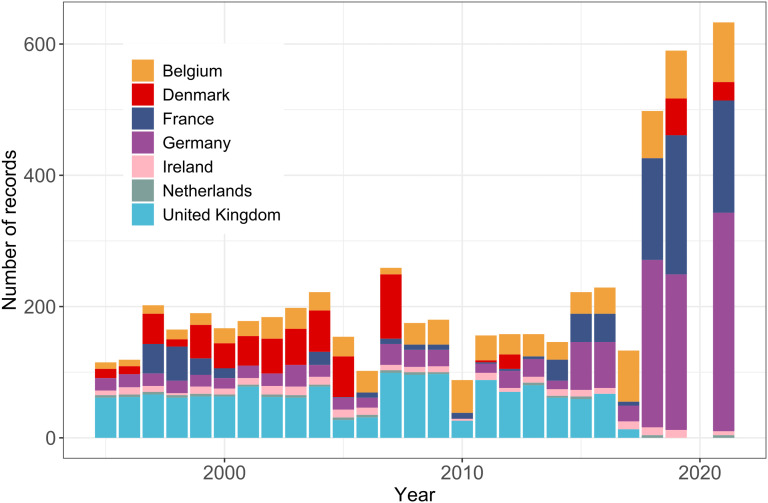
Annual number of port dredging records by country (colour scale) from EMODnet (1995-2021).

**Fig 3:**
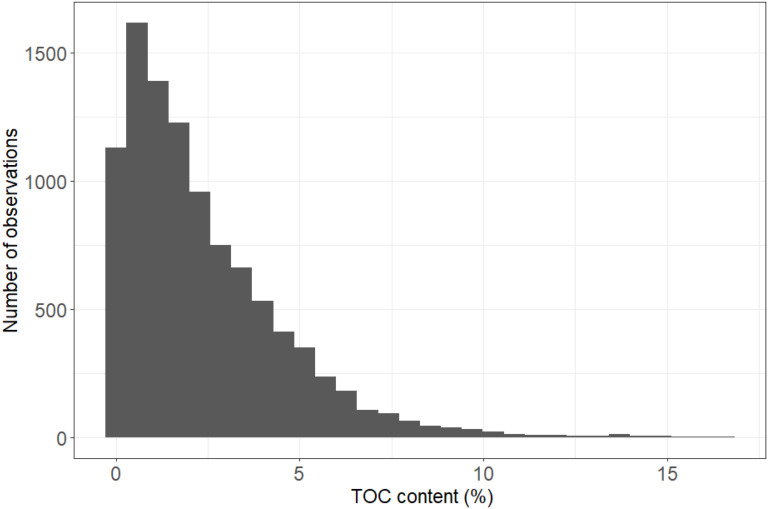
Percentage total organic carbon content in coastal and inshore areas. %TOC content of sediment samples within 5 km of the coastline across the Northwest European Shelf (median = 1.78%, 5^th^ percentile = 0.11%, 95^th^ percentile = 6.46%)**.**

**Fig 4:**
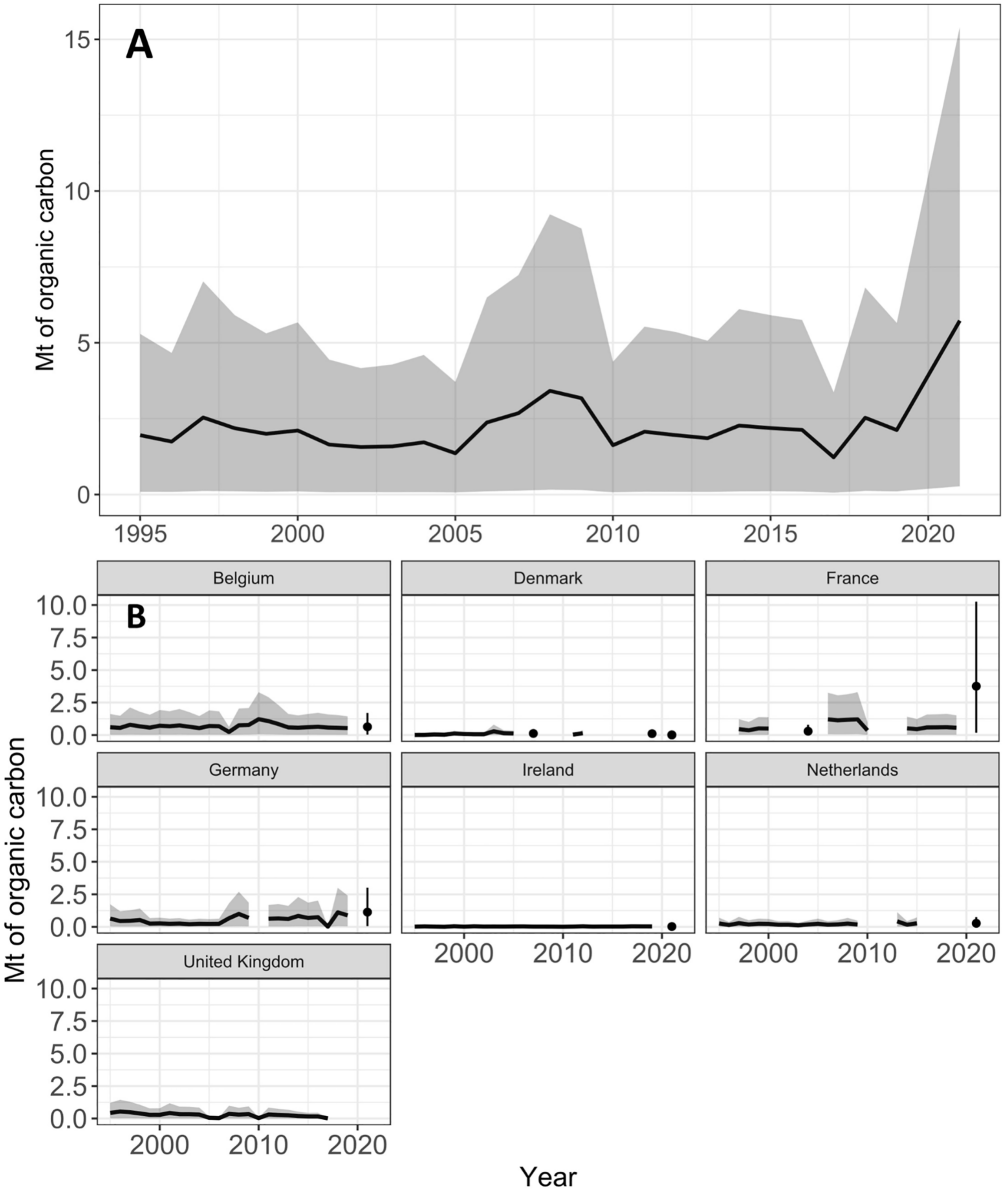
Estimated sedimentary organic carbon disturbance (Mt) from port dredging between 1995 and 2021. A) Annual mean estimates of carbon disturbed by port dredging across the Northwest European Shelf (solid line), with upper and lower estimates (shaded grey area) based on 5^th^ and 95^th^ percentile ranges of simulation results. B) mean estimates of carbon disturbance annually in individual countries (solid line, years with no data in adjacent years for presentation purposes), with upper and lower estimates made with 5^th^ and 95^th^ percentiles (shaded grey area, vertical lines for years with no adjacent data).

Some aggregate extraction records prior to 1991 lacked information on the proportions of sediment types extracted each year. In addition, annual totals of extracted sediment were reported only at the national scale, without specifying the quantities from individual extraction areas (meaning sediment type could not be inferred from substrate maps). For these records, %TOC values were randomly sampled from a combined distribution representative of sand, coarse, and mixed grained sediments. This step was taken as only a few specific substrate types are targeted for marine aggregate extraction, due to end use requirements in the construction industry. As a result, %TOC contents in target sediments are similar to each other, particularly relative to muddy sediments in coastal settings. To validate this, we investigated how the shelf wide estimate of carbon disturbance from aggregate extraction in 1980 changed when we varied the assumed proportion of sediment types extracted (S3 Fig).

We applied the approach to generate both country-level and shelf-wide annual estimates of mass of carbon disturbed (in megatons, Mt). Spatial analyses were carried out in QGIS (Version 3.32.3) with figures produced in R using the packages ggplot2 and cowplot [47,48].

## Results

### Port and harbour dredging

At the shelf scale, a total of 6332 port dredging records were extracted from EMODnet, spanning 1995–2021 (Fig 1). Records were significantly more numerous between 2018 and 2021, apart from in 2020, most likely as a result of the COVID-19 pandemic. Many (47%) of the available records between 2018 and 2021 were associated with ports in Germany (Fig 2). Prior to this period, the largest proportion of records were from ports in the UK and Denmark. Additionally, 135 records of dredging at UK ports were collated from historical sources for the years 1835–1845 and 1883–1903, although these records were in 10- and 20-year summaries, respectively.

Approximately 95% of the port and harbour dredging sites were situated less than 2.5 km from shore. The total reported amount of sediment removed annually from these sites ranged from 52 to 246 Mt year^-1^ between 1995 and 2021 (S4 Fig), with estimated mean sediment removal during this period of 93.7 ± 37.2 Mt year^-1^ (results are reported in text as mean ± 1 standard deviation throughout).

Total organic carbon content in coastal and inshore sediment samples ranged widely, from 0.01% up to 16.56% (Fig 3). This resulted in a ‘most likely’ estimate of organic carbon disturbance from port dredging across the Northwest European Shelf ranging between 1.2 ± 1.1 Mt at its lowest in 2017 and 5.7 ± 5.2 Mt of organic carbon, at its highest in 2021 (Fig 4A). Across all years examined, mean annual carbon disturbance was estimated at 2.2 ± 0.9 Mt of organic carbon.

**Fig 5:**
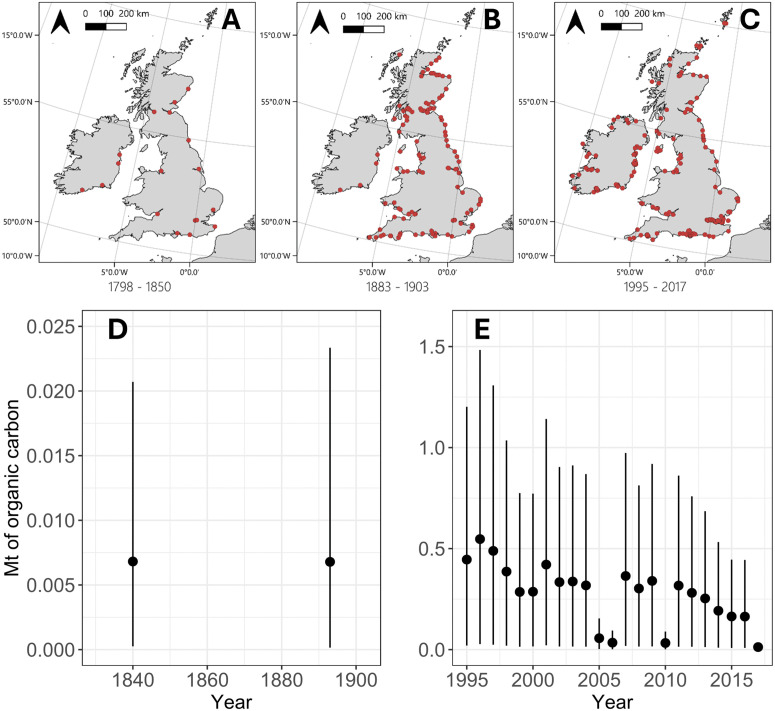
A-C) Spatial distribution of port and harbour dredging sites across the UK and Ireland, from sources in Table 1. The 200m is denoted by the thin black line. D) Annual estimates of sedimentary organic carbon disturbance by port dredging in the UK during the 19^t^^h^ century, where vertical error bars represent the 5^th^ and 95^th^ percentile ranges. As historical data for the 19^th^ century were provided in 10- and 20-year summaries, the data points for 1840 and 1893 reflect estimated annual mean disturbance calculated for the periods 1834-1845 and 1883-1903, respectively. E) Annual estimates of sedimentary organic carbon disturbance by port and harbour dredging between 1995 and 2017. Note differing y-axis scales in panels D and E. The basemap in this figure is in the public domain (Available from https://www.naturalearthdata.com/). Projection: LAEA Europe EPSG: 3035.

Historical records for UK waters, describing port dredging activities in the 19^th^ century, allowed for insights of carbon disturbance from port dredging over centennial timescales. Our ‘most likely’ estimate of disturbance from port dredging between 1834–1845 was 0.07 Mt of organic carbon over a ten-year period, with ~0.1 Mt of organic carbon disturbed over the 20 years between 1883–1903. To better interpret sparse data points, we further investigated the spatial distribution of port dredging activities across the UK and Ireland between 1798, the first qualitative mention of dredging in the UK [24], and 2017, when the last available UK port dredging records were available from EMODnet (Fig 5 A-C). This revealed that the number of dredging sites in the UK and Ireland markedly increased from 18 to 117 during the latter half of the 19^th^ century.

### Marine aggregate extraction

**Fig 6:**
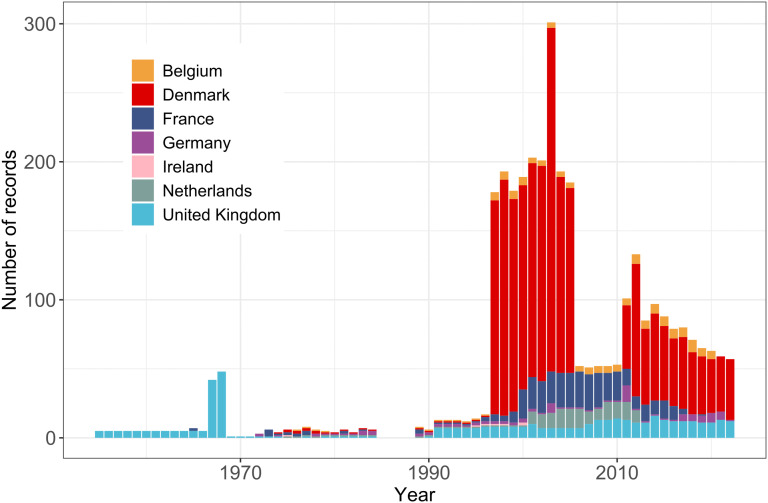
Aggregate extraction records by country (1955-2022), compiled from public records, published literature, and EMODnet.

A total of 3405 marine aggregate extraction events were used to estimate organic carbon disturbance between 1955 and 2022. The earliest marine aggregate extraction records originated from UK waters in 1955, while data from other countries were available after 1972. From 1972 onwards, countries such and France and Denmark contributed a large proportion of records (Fig 6).

Marine aggregate extraction sites were found further offshore, relative to port and harbour dredging sites, with ~75% of licenced areas situated over 7.5 km from shore. Based on our records, the mean annual volume of sediment extracted on the Northwest European Shelf between 1955 and 2022 was 27.4 Mt year^-1^ and ranged between 3.2–96.9 Mt year^-1^. Averaged over the whole study period, approximately 53% of material extracted from these sites consisted of sands, 46% of coarse-grained sediment and less than 1% of mixed sediments (S5 Fig).

**Fig 7:**
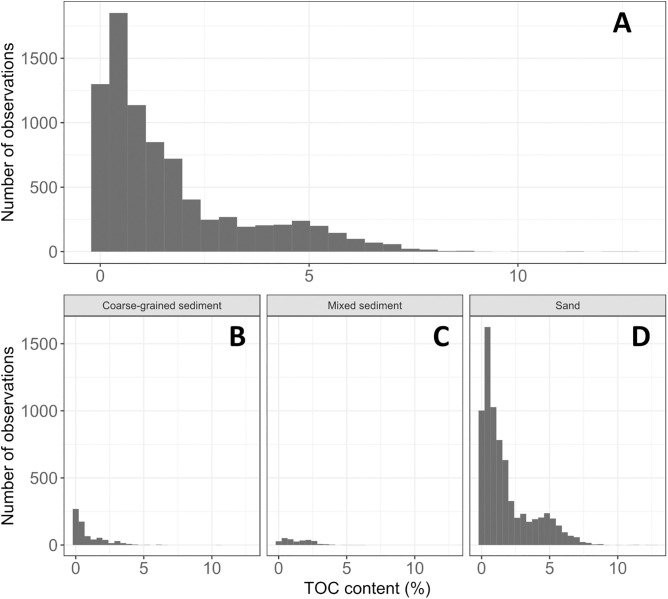
A) Percentage total organic carbon content (%TOC) in aggregate extraction sites on the NWES (median = 0.48%, 5^th^ percentile = 0.04%, 95^th^ percentile = 5.19%). B-D) %TOC content of shelf sediments plotted in panel A, separated by sediment type.

**Fig 8:**
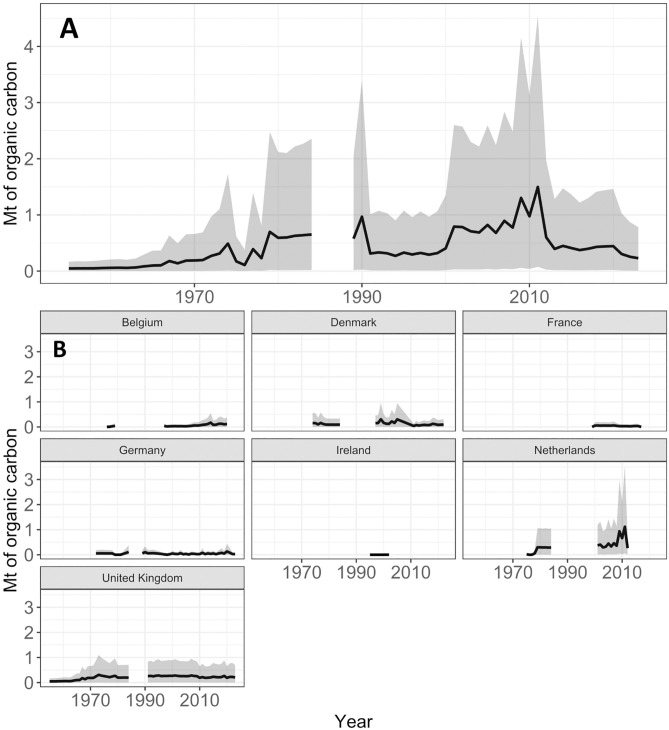
A) Annual estimates of sedimentary organic carbon disturbance by marine aggregate extraction across the Northwest European Shelf (solid line), with 5^th^ and 95^th^ percentile range estimates (grey shaded area). B) mean estimates of carbon disturbance annually in individual countries (solid line), with upper and lower estimates made with 5^th^ and 95^th^ percentiles (shaded grey area).

Total organic carbon content in sediments of the same type as those in found in aggregate extraction sites ranged from 0.0% to 12.67%, with a median of 0.48% (Fig 7). In terms of organic carbon disturbance in these areas, we estimated that aggregate extraction activities impact between 0.04 ± 0.05 (in 1955) and 1.5 ± 0.7 (in 2011) Mt organic carbon year^-1^ on the shelf (Fig 8A), with a mean annual disturbance of 0.4 ± 0.3 Mt organic carbon year^-1^ (1955−2022). Despite data gaps in our timeline (e.g., between 1985−88), carbon disturbance appeared to gradually increase through the second half of the 20^th^ century, peaking at 1 Mt organic carbon year^-1^ in 1990, and increasing further to 1.5 Mt organic carbon year^-1^ in 2012. However, in more recent years (2012–2022) carbon disturbance from aggregate extraction appears to have declined and has remained less than 0.5 Mt organic carbon year^-1^ (Fig 8A).

At the national scale, the UK has maintained relatively stable, and generally higher, levels of carbon disturbance since the 1960s compared with most other countries (Fig 8), which aligns with its role as an exporter of aggregates. In comparison, Dutch aggregate extraction commenced later but, despite some data limitations, reached the highest organic carbon disturbance rates among all countries considered here, exceeding 1 Mt organic carbon year ⁻ ¹ in 2012. Mean annual carbon disturbance by country followed a descending order of: Netherlands, UK, Denmark, France, Belgium, Germany, Ireland.

## Discussion

Previous research on seabed organic carbon disturbance has focused largely on bottom trawling, leaving the impacts of other marine industries, such as aggregate extraction and port dredging, poorly understood. Our analysis provides the first, first-order, estimates of organic carbon disturbance on the Northwest European Shelf arising from seabed dredging of ports and harbours, and by the marine aggregate extraction industry. Our mean estimate of disturbance from port dredging was 2.2 ± 0.9 Mt organic carbon year^-1^ across the study area between 1995 and 2021. Furthermore, analysis of archival documents indicates that mechanised dredging at UK ports in the ten years between 1834−1845 disturbed an estimated 0.07 Mt organic carbon, increasing to 0.1 Mt across 1883−1903. We estimate that carbon disturbance from aggregate extraction had a mean value of 0.4 ± 0.3 Mt organic carbon year^-1^ between 1955 and 2022. Estimates for all years have wide uncertainty ranges, with lower bounds approaching zero, reflecting variability in the potential organic carbon content in extracted sediments. A potential contributing factor to this wide range of organic carbon values may come from the natural variability encompassed by a large dataset, and the variability between different methods used for determining the organic carbon content of marine sediments [49].

### Port and harbour dredging

Existing models predicting organic carbon content across the Northwest European continental Shelf exhibited a poor spatial overlap with European port and harbour dredging activities [1,33]. We found that 95% of dredging activities occurred within 2.5 km of the coastline, an area that is poorly resolved in shelf-scale carbon stock models of the Northwest European Shelf. It is important to note that coastal sediments are generally enriched in carbon relative to outer-shelf sediments, particularly close to estuaries, and so using a model primarily trained on samples from shelf sediments, is likely to lead to an underestimation of the mass of carbon disturbed by dredging activities [34]. In this study, we utilised coastal carbon measurements from across the Northwest European Shelf, by filtering the EURO-CARBON data set published by Graversen et al. [46].

Historical records from the UK revealed that the spatial distribution of carbon disturbance increased greatly across the UK coastline during the 19^th^ century. Written records from harbour authorities in the UK between 1883 and 1903 indicate that eight ports were dredged annually in this period, with most regular dredging occurring at Aberdeen, the Clyde, and Ramsgate. The observed increase in dredge sites after 1903 may be explained by greater access to dredgers, but also potentially by rises in siltation rates caused by land clearance for agriculture [50]. Port dredging increased over the 20^th^ century, particularly between the 1940s and 1980s, driven by the need to accommodate larger ship drafts and maintain deeper navigational channels [51]. However, this increase was not represented in our estimates as it fell within a large data gap of available records (Table 1). Within the UK total estimated disturbance in the 21^st^ century, ~ 3.8 Mt of organic carbon (totalled between 2000–2017), showed a near 30-fold increase relative to the total estimated carbon disturbance in the 20 years at the end of the 19^th^ century (~0.1 Mt of organic carbon, totalled between 1883–1903).

Across the wider Northwest European Shelf in the late 20^th^ and 21^st^ centuries, organic carbon disturbance at ports was highest in Belgium and Germany from 1995 onwards. Both countries have numerous large seaports situated along estuaries, which require numerous dredge sites to maintain navigational depths. Compared to ports on the open coast, these environments experience high rates of sedimentation and are dependent on continuous one-way access channels that can reach 40–60 nautical miles inland [52]. In most cases, estuarine dredging sites appeared further upstream than sediment samples within the available database [46], however other published case studies from the Thames and Seine estuaries [53,54] report maximum %TOC contents of ~14% and 7.3% respectively. These values are lower than the maximum used in our distribution of potential %TOC content at port and harbour dredging locations (16.6%) [46]. This suggests that the range of values used to estimate organic carbon disturbance at port dredging locations are appropriate, and inclusive of these upstream dredging sites where %TOC content is expected to be highest. Due to a lack of published data describing TOC contents in port and harbour sediments, a previous study investigating the organic carbon impacts of dumping dredged sediments [55] applied an assumed TOC content range of 0–8% to all North Sea based dredged material. This was derived from measurements from a single port (Hamburg). In contrast, our wider maximum %TOC content range (0–16.6%, where 95% of sampled data falls below 6.5%) is based on a comprehensive dataset of samples from across the study area (n = 9920). This approach aims to be more representative of regional variability by including port areas that contain higher %TOC contents, such as the Thames and the Seine estuaries [53,54], which are often underrepresented in shelf wide averages.

Aside from illustrating differences at the national level in the 21st century, our findings provide evidence of a gradual increase in carbon disturbance from port dredging across the Northwest European Shelf between 1995 and 2021, punctuated by years of higher extraction activity. Carbon disturbance was relatively constant for large parts of the timeline, with a most years seeing a disturbance of ~ 2–2.5 Mt organic carbon year^-1^ across all ports. However, substantial peaks above these values are seen in 2008–2010 and 2021. While the drivers of these fluctuations are not fully understood, they may relate to large port development projects or major dredging campaigns. For example, the large increase in organic carbon disturbance in France (2021) may be due to numerous port development projects commencing, including construction of new quays at Dunkirk and La Nouvelle, and connecting the container area at Le Havre to the Seine system of port basins [56]. Observed short-term variability is likely related to the fact maintenance dredging works are not ubiquitously undertaken on an annual basis, as well as potential inconsistencies in the frequency and quality of data collection or reporting.

Periodic sediment removal through maintenance dredging has been cited as a reason port and harbour sediments cannot be considered long-term sedimentary carbon stores [57]. While dredging regularly disturbs port and harbour systems at some sites [58], the sediments within these systems still accumulate and store organic carbon derived from both terrestrial and marine primary production [59]. As a result, we consider sediment removal by dredging as a ‘disturbance’, defined as a mechanical intervention that re-exposes buried organic carbon to the water column, regardless of the systems history. This is supported by the potential for physical disturbance to enhance remineralisation rates through mixing and reoxygenation [55,60], particularly in carbon-rich sediments, such as those found in coastal and inshore areas [34].

From a management perspective, the assessment of port dredging as a ‘disturbance’ to marine sediments raises an important question regarding baseline disturbance levels. Since most ports and harbours are engineered systems that require regular maintenance, it could be argued that dredging is a normal function of the site rather than a disruption of a stable environment. However, defining undisturbed baselines is highly challenging in marine management, particularly in cases where areas have been chronically modified for several centuries (e.g., in areas of the North Sea that have been trawled for several centuries). While carbon accumulation in ports may differ from other long-term sequestration sinks due to regular dredging, these sites contain significant pools of terrestrial and marine-derived carbon. As dredging prevents the natural burial of this material, placing a proportion of the organic matter at risk of remineralisation, quantifying these masses involved is valuable to distinguish how anthropogenic activities differ in their potential impacts to sedimentary organic carbon in marine environments.

Additional impacts of these anthropogenic extractive activities on organic carbon stores in continental shelf sediments may occur through resuspension, redeposition, and smothering during offshore disposal of dredged material [55]. We did not analyse sediment dumping in this study due to uncertainties in sediment transport dynamics and biogeochemical characteristics of deposited material, although this could be a valuable area of future research.

### Marine aggregate extraction

The median organic carbon content of sediments targeted by aggregate extraction was considerably lower (0.48% TOC) than that of port dredging (1.78% TOC). This nearly 4-fold difference is related to the targeting of coarse-grained sediments by the aggregate extraction industry, which store less carbon than finer sediments such as mud [61]. Our estimated typical shelf sediment TOC content is of the same order of magnitude as, although slightly lower than, the mean TOC concentration for North Atlantic continental shelf sediments (0.63%) reported in a recent global model [62]. The results presented here suggest significant increase in organic carbon disturbance from marine aggregate extraction across the Northwest European Shelf between 1955 (0.05 ± 0.05 Mt organic carbon year^-1^) and 2021 (1.5 ± 0.7 Mt organic carbon year^-1^). In a similar study across the North Sea, Porz et al. [55] estimated that between 0.1 and 0.2 Mt of organic carbon are present in sediments extracted annually by the marine aggregate industry. While their study overlaps with 55% of our study sites, their findings are of the same order of magnitude as the results presented here and align with our mean annual disturbance estimate of 0.4 ± 0.3 Mt of organic carbon year^-1^ by marine aggregate extraction.

Review of archival records from the UK suggest that commercial marine aggregate extraction has occurred since the 1880s. In addition, the industry underwent a significant expansion during the 1980s due to growing demand for construction materials and coastal development [44]. Specifically, we see large peaks in disturbance during the 1980s and again in the early 2000s, which may be linked to general trends in economic growth in Europe or large construction projects being supported by UK exports of marine aggregates [44]. In the interim period during the 1990s, the demand for sand-based aggregates is known to have stabilised in northern Europe [63], a trend which is partly reflected in our estimates of carbon disturbance rates over that time. However, our data deviated from this trend at around 1990, when we observe a pronounced decrease in estimated carbon disturbance, followed by a sharp increase between 1999 and 2001. This apparent drop-off around 1990 likely reflects inconsistencies in EMODnet records, as major present-day contributors like the Netherlands and Denmark did not supply data for the 1990s.

At the country level, UK and the Netherlands disturb the greatest masses of organic carbon. In the UK, a steady increase in disturbance is seen after the 1960s, peaking in 1995 (~0.3 Mt). Elevated disturbance in the UK relative to other countries results from larger masses of sediment removal, rather than disturbance of particularly carbon-rich sediments. This is evidenced by the nearly 4-fold lower %TOC content of sediments targeted for aggregate extraction, but relatively higher marine aggregate production rates to meet the UK’s role as an exporter of marine gravels [44]. Disturbance in the Netherlands rose sharply from the 1970s to a peak in 2011 (~0.9 Mt organic carbon year ⁻ ¹), which likely reflects extensive offshore extraction to support construction and coastal protection. Other countries such as Belgium, France, and Germany contributed intermittently and at lower magnitudes (<0.1 Mt organic carbon year⁻^1^). Potential reasons for this may include the import of marine gravels from the UK in areas that lack exploitable gravel deposits, such as Belgium, or the aggregate demands of France being met by terrestrial and fluvial sources [44].

Our results show slightly lower levels of sediment removal by aggregate extraction industries than port dredging, with mean annual removal estimated at 27.4 Mt year^-1^ and 93.7 Mt year^-1^, respectively. When considered with the lower %TOC content of sediments targeted for aggregate extraction, relative to the %TOC content of sediments impacted by port dredging, these two factors explain why estimated masses of organic carbon disturbance from aggregate extraction industries are marginally lower than estimates of carbon disturbance by port dredging shelf wide.

### Limitations

Given that there is a large amount of uncertainty in how best to estimate CO_2_ emissions from anthropogenic sediment disturbance [13,14,17], we have opted to estimate the masses of organic carbon disturbed by extractive industries, rather than the amount that was remineralised and emitted as CO_2_. The proportion of this disturbed organic carbon that is subsequently put at risk of remineralisation, and potential emission, is a function of multiple variables. These include the concentration and reactivity of organic matter within the sediment, dictated by biological, geochemical, and physical environmental factors, as well as the total oxygen exposure time, which is often mediated by natural or anthropogenic physical disturbance [64,65]. This approach was considered appropriate given the considerable uncertainties surrounding the biogeochemical fate of disturbed sedimentary carbon and its varying vulnerability to remineralisation under different environmental conditions. It ensures that our estimates are as robust as possible within the limits of current knowledge. In addition, data gathered and analysed for this study carry some inherent uncertainties.

For example, the port dredging data held by EMODnet do not cover all dredging events on the Northwest European Shelf. The database is the aggregation of data voluntarily provided by specific countries and stakeholders, and therefore our estimates of dredging induced carbon disturbance are likely underestimates. Specifically, dredging data relating to navigational channels are not provided by all countries, and similarly, data for smaller ports and harbours are frequently omitted. For instance, the Dutch data only represents activities at seven large seaports, despite 19 ports being listed for the Netherlands in the World Port Index [https://msi.nga.mil/Publications/WPI]). Conversely, data provided for German waters includes numerous estuarine and riverine navigational dredge sites for commercial ports upstream, which are generally not provided by other countries. Furthermore, the highly coastal and estuarine nature of port dredging means that missing data will be in areas characterised by fine muds and higher concentrations of organic carbon [34,66,67]. The potential scale of these missing data can be illustrated by comparing our data to those reported by OSPAR between 2008–2020. Total annual dredging volumes are aggregated and reported over the OSPAR region (the northeast Atlantic area, i.e., including countries such as Iceland, Norway, Spain and Portugal). EMODnet provides data over the same area for the period 2008–2020 and are consistently between half and two thirds of the values reported by OSPAR [68]. However, the OSPAR dredging data are only provided as regional totals, and are not spatially explicit at a national level, meaning that we were unable to include these data in our analysis due to a mismatch between the countries contributing to OSPAR data, and countries within our study area. Dredged sediment disposal data are also reported by OSPAR [68] and are spatially explicit at a national level. However, as not all dredged sediment is dumped at sea, and disposal records do not account for sediment diverted for landfill or re-use, these records are unsuitable to accurately represent initial extraction volumes required for this analysis.

Similarly, our estimates of organic carbon disturbance by marine aggregate extraction are also likely conservative. A key reason for this is that marine aggregates are typically extracted using a screening process, where unwanted sediment grain sizes are removed and returned to the seabed [69]. As a result, a much greater volume of sediment is disturbed during aggregate extraction than is retained as ‘landed cargo.’ Our estimates of organic carbon disturbance are based on these retained masses, so a greater amount of sediment and thus carbon would have been disturbed where screening is common practice. Additionally, we were unable to determine the proportion of different sediment types comprising extracted marine aggregates prior to 1991 due to insufficient information within records, introducing additional uncertainty in the estimated %TOC content of these sediments.

Finally, some inconsistencies exist between the amounts of aggregate extraction in the reports produced by ICES [70] (one annual total, not broken down by sediment type) and the values of amounts extracted available on EMODnet (amounts extracted from individual licence areas). For example, extraction in France in 2005 reported by EMODnet is approximately half of the extraction reported by ICES in the same year. These inconsistencies have the potential to under or over represent organic carbon disturbance by individual countries. Using French aggregate extraction in 2005 as an example, the ‘most likely’ carbon disturbance using extraction data from EMODnet is 0.05 Mt of organic carbon, and 0.2 Mt of organic carbon using extraction data from ICES. This supports previous studies that have also observed inconsistencies within data provided by EMODnet, describing offshore oil and gas infrastructure [71].

With growing interest in marine sediments and their carbon storage benefits, including sediment-targeting extractive industries in carbon stock assessments is important for comprehensive management plans. This finding of inconsistencies in reports of aggregate extraction activities demonstrates the need for a reliable source of data describing where, how much, and what types of sediments are being extracted. To further increase the accuracy of estimated impacts to sedimentary organic carbon from anthropogenic activities and understand how these potentially translate to subsequent CO_2_ emissions, there is a need for a stronger understanding of the organic carbon content of extracted sediments, its reactivity, and accurate dry bulk density measurements [72]. Improving the latter would reduce the uncertainty in our results associated with converting extracted sediment volumes into masses using an average density estimate based on all sediment classes.

### Comparisons with other industries

Recent research on organic carbon disturbance by the bottom trawl fishing industry in UK waters [17] allows us to contextualise our findings across other marine industries. Previous research has estimated that sediments impacted annually by bottom trawling within the UK exclusive economic zone contain ~109 Mt of organic carbon based on fishing data between 2009 and 2018 [19]. Between 2009 and 2018 our ‘most likely’ estimates of disturbance by aggregate extraction ranged between 0.2 ± 0.1 Mt organic carbon year^-1^ and 0.3 ± 0.1 Mt organic carbon year^-1^ across the same area, with a mean estimate of 0.2 ± 0.02 Mt organic carbon year^-1^. For UK port dredging, in the same period, ‘most likely’ disturbance estimates had a maximum 0.34 ± 0.31 Mt organic carbon year^-1^, with a mean of 0.2 ± 0.1 Mt organic carbon. Therefore, our results suggest annual sedimentary carbon disturbance associated with bottom trawling within the UK exclusive economic zone is potentially three orders of magnitude higher than that of disturbance by port dredging and aggregate extraction in the same area.

## Conclusions

This study provides first-order estimates of continental shelf-wide seabed organic carbon disturbance from port dredging and marine aggregate extraction in European waters. These industries pose notable disturbance risks to shelf wide carbon stores, but disturbance levels within the UK appear to be roughly three orders of magnitude lower than that of bottom trawling. While spatial management strategies to protect seabed organic carbon from trawling could be relatively straightforward to introduce, organic carbon protection is much more difficult to enact for the port dredging industry, due to the fixed nature of ports and harbours and the necessity for safe navigation. Marine aggregate extraction, generally targets sediments low in organic carbon, reducing its potential for impacts compared to industries that disturb carbon-rich muds. This work underscores the need to assess the impacts of seabed-disturbing industries on sedimentary carbon stocks, thereby supporting more effective management of shelf seas.

## Supporting information

S1 Table Information about the cost of dredging works and the mass of sediment extracted during dredging works, from 6 ports prior to 1903. (DOCX)

S2 Table Estimated masses of sediment removed during dredging works prior to 1903, based on the cost of the works listed in historical sources. (DOCX)

S1 Fig Map of available cores from the EURO-Carbon database within the study area [46]. Graversen et al., 2025. A marine and salt marsh sediment organic carbon database for European regional seas (EURO-CARBON). Data in Brief 60, 111595. https://doi.org/10.1016/j.dib.2025.111595. The basemap in this figure is in the public domain (available from https://www.naturalearthdata.com/). Projection: LAEA Europe EPSG: 3035.
(TIFF)

S2 Fig Changes to mean estimates of carbon disturbance in Belgium in 1995 (points) from Monte Carlo simulations with varying numbers of runs. For each number of runs, 10 independent iterations were made to generate an estimated mean output, and standard deviation between iterations (error bars).
(TIF)

S3 Fig Estimated organic carbon disturbance from marine aggregate extraction in 1980, dependent on the percentage of extracted sediment assumed to be sand vs coarse-grained sediments. (TIF)

S4 Fig The amount of sediment removed annually by port and harbour dredging on the Northwest European Shelf between 1995 and 2021. (TIF)

S5 Fig The amount of sediment removed annually by marine aggregate extraction on the Northwest European continental shelf between 1955 and 2022. (TIF)
