# Supplementary material for: Estimating historic seabed carbon disturbance by port dredging and aggregate extraction in NW Europe
Source: PLoS One. 2026 May 27;21(5):e0349191. doi: 10.1371/journal.pone.0349191 (PMC13215537; doi:10.1371/journal.pone.0349191)
Supplement: S1 Table — (DOCX) [file pone.0349191.s001.docx]

| **Port** | **Date** | **kg removed** | **Cost** | **Cost / kg** |
| --- | --- | --- | --- | --- |
| Ramsgate | 1900 | 81280000 | 3520 | 0.0000433071 |
| Torquay | 1900 | 73000000 | 5,500 | 0.0000753425 |
| Ipswich | 1814 | 22100000 | 1105 | 0.00005 |
| Aberdeen | 1900 | 5.77E+09 | 112,974 | 0.0000195891 |
| Wear | 1900 | 6.1E+09 | 84370 | 0.0000138318 |
| Yarmouth | 1831 | 2.13E+08 | 2,387 | 0.0000112112 |
| Arbroath | 1834-1845 | 43217228 | 8993 | 0.000208088 |
