# Supplementary material for: Estimating historic seabed carbon disturbance by port dredging and aggregate extraction in NW Europe
Source: PLoS One. 2026 May 27;21(5):e0349191. doi: 10.1371/journal.pone.0349191 (PMC13215537; doi:10.1371/journal.pone.0349191)
Supplement: S2 Table — (DOCX) [file pone.0349191.s002.docx]

| **Port** | **Cost** | **Estimated kg removed** | **Date** |
| --- | --- | --- | --- |
| Aberdeen | 5648.7 | 93838929 | 1883-1903 |
| Alloa | 864.05 | 14354015 | 1883-1903 |
| Banff | 75 | 1245936 | 1883-1903 |
| Berwick | 145 | 2408810 | 1883-1903 |
| Bridgeness | 50 | 830624.1 | 1883-1903 |
| Bridlington | 853 | 14170447 | 1883-1903 |
| Bristol | 12357 | 205000000 | 1883-1903 |
| Cambeltown | 322.95 | 5365001 | 1883-1903 |
| Castletown | 235 | 3903933 | 1883-1903 |
| Charlestown | 334 | 5548569 | 1883-1903 |
| Clyde | 7065.8 | 117000000 | 1883-1903 |
| Conwy | 10 | 166124.8 | 1883-1903 |
| Cullen | 236 | 3920546 | 1883-1903 |
| Dartmouth | 150 | 2491872 | 1883-1903 |
| Dundee | 4350 | 72264298 | 1883-1903 |
| Eyemouth | 1083 | 17991318 | 1883-1903 |
| Folkestone | 6000 | 99674894 | 1883-1903 |
| Fraserbrugh | 1913 | 31779679 | 1883-1903 |
| Grangemouth | 5261.25 | 87402422 | 1883-1903 |
| Granton | 1389.5 | 23083044 | 1883-1903 |
| Hartlepool | 1,797 | 29852631 | 1883-1903 |
| Helensburgh | 962 | 15981208 | 1883-1903 |
| Helmsdale | 206.3 | 3427155 | 1883-1903 |
| Ilfracombe | 1810 | 30068593 | 1883-1903 |
| Inverness | 953.5 | 15840002 | 1883-1903 |
| Kings Lynn | 790 | 13123861 | 1883-1903 |
| Kirkcaldy | 81 | 1345611 | 1883-1903 |
| Lancaster | 8340 | 139000000 | 1883-1903 |
| Lossiemouth | 1000 | 16612482 | 1883-1903 |
| Lowesoft | 5000 | 83062411 | 1883-1903 |
| Montrose | 382.5 | 6354274 | 1883-1903 |
| Newlyn | 1000 | 16612482 | 1883-1903 |
| Neyland | 5657 | 93976812 | 1883-1903 |
| North Sunderland | 654 | 10864563 | 1883-1903 |
| Penarth | 750 | 12459362 | 1883-1903 |
| Penryn | 1240 | 20599478 | 1883-1903 |
| Penzance | 4961 | 82414525 | 1883-1903 |
| Plymouth | 8500 | 141000000 | 1883-1903 |
| Poole | 1350 | 22426851 | 1883-1903 |
| Port Gordon | 955 | 15864921 | 1883-1903 |
| Porthmahomack | 57 | 946911.5 | 1883-1903 |
| Ramsgate | 3520 | 58475938 | 1883-1903 |
| Sandwich | 998 | 16579257 | 1883-1903 |
| Scarborough | 3820 | 63459682 | 1883-1903 |
| Severn | 2000 | 33224965 | 1883-1903 |
| Southampton | 3468.75 | 57624548 | 1883-1903 |
| St Monance | 100 | 1661248 | 1883-1903 |
| Stirling | 198 | 3289271 | 1883-1903 |
| Stornoway | 240.75 | 3999455 | 1883-1903 |
| Stranraer | 534 | 8871066 | 1883-1903 |
| Sutton | 914.25 | 15187962 | 1883-1903 |
| Teignmouth | 252.6 | 4196313 | 1883-1903 |
| Thames | 1512.75 | 25130533 | 1883-1903 |
| Torquay | 5500 | 91368653 | 1883-1903 |
| Wear | 4218.5 | 70079757 | 1883-1903 |
| Whitby | 18182 | 302000000 | 1883-1903 |
