# Supplementary figures and images for: Estimating historic seabed carbon disturbance by port dredging and aggregate extraction in NW Europe
Source: PLoS One. 2026 May 27;21(5):e0349191. doi: 10.1371/journal.pone.0349191 (PMC13215537; doi:10.1371/journal.pone.0349191)

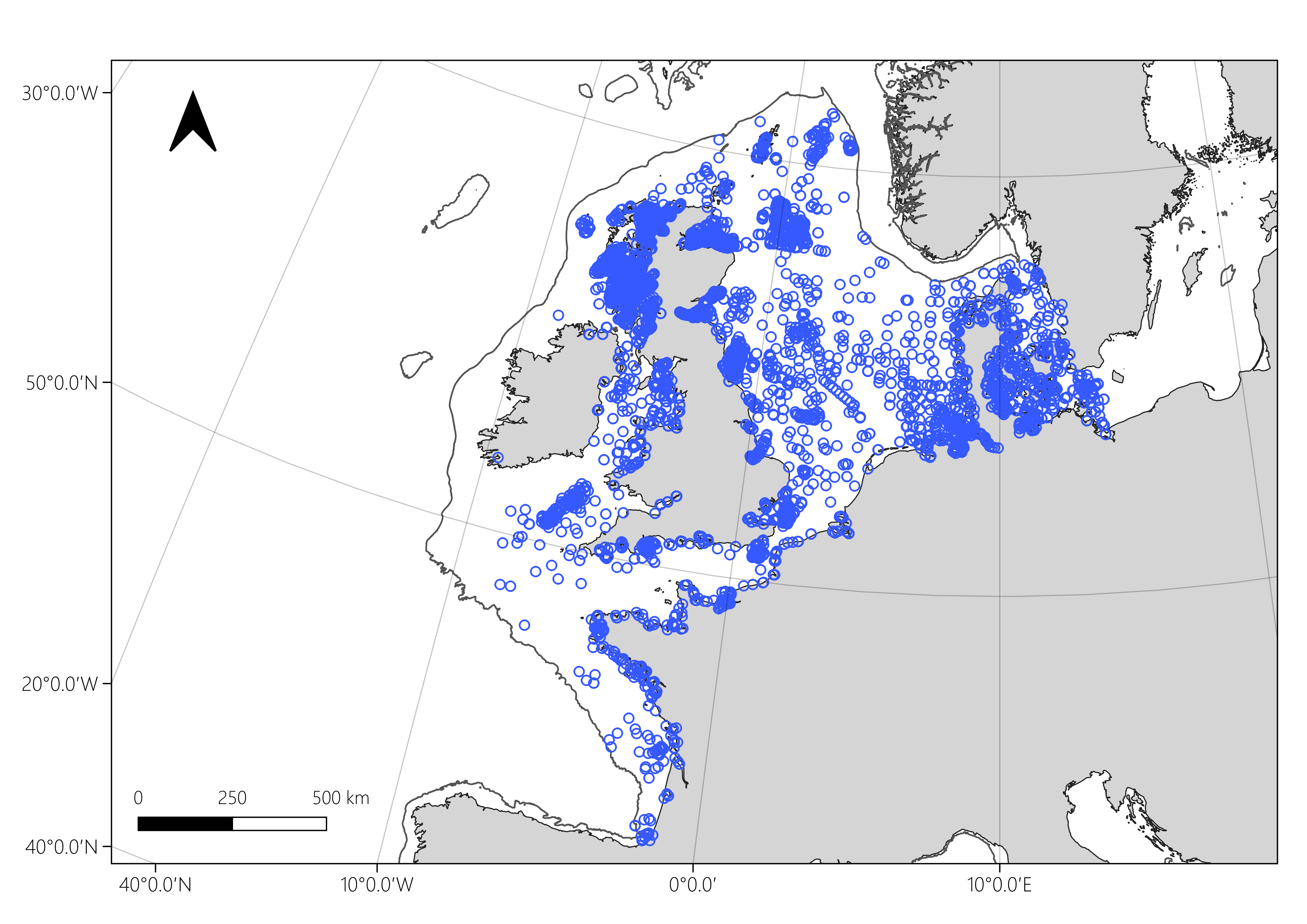

Supplement: S1 Fig — Graversen et al., 2025. A marine and salt marsh sediment organic carbon database for European regional seas (EURO-CARBON). Data in Brief 60, 111595. https://doi.org/10.1016/j.dib.2025.111595. The basemap in this figure is in the public domain (available from https://www.naturalearthdata.com/). Projection: LAEA Europe EPSG: 3035. (TIFF) [file pone.0349191.s003.tiff]

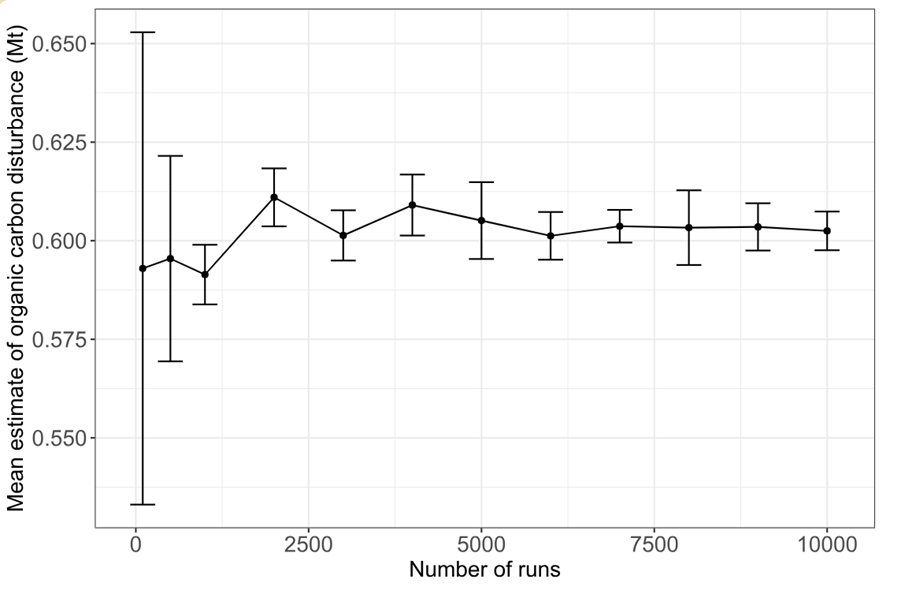

Supplement: S2 Fig — For each number of runs, 10 independent iterations were made to generate an estimated mean output, and standard deviation between iterations (error bars). (TIF) [file pone.0349191.s004.tif]

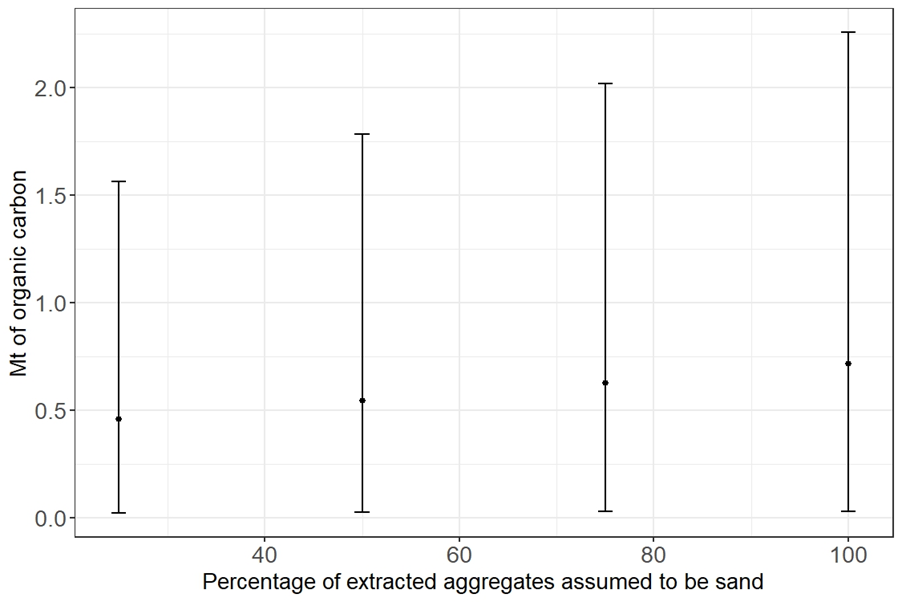

Supplement: S3 Fig — (TIF) [file pone.0349191.s005.tif]

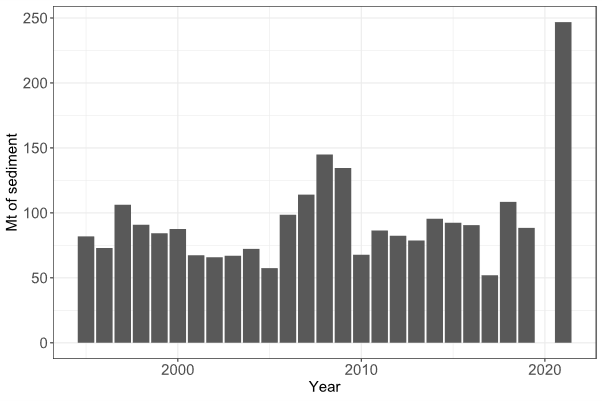

Supplement: S4 Fig — (TIF) [file pone.0349191.s006.tif]

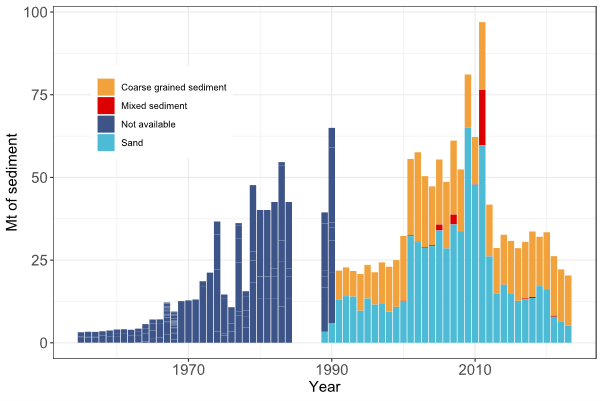

Supplement: S5 Fig — (TIF) [file pone.0349191.s007.tif]
